# Supplementary material for: Red Beetroot’s NMR-Based Metabolomics: Phytochemical Profile Related to Development Time and Production Year
Source: Foods. 2021 Aug 15;10(8):1887. doi: 10.3390/foods10081887 (PMC8393249; doi:10.3390/foods10081887)
Supplement: Supplementary file 1 [file foods-10-01887-s001.zip › foods-1308059-supplementary.pdf]

Red beetroot's NMR-based metabolomics: phytochemical profile related to development time and production year

Fabio Sciubba [1,2§], Ottavia Giampaoli [1,2§], Giorgia Conta [1,2], Giorgio Capuani [1,2], Alberta Tomassini [1,2], Giorgio Giorgi [3], Elisa Brasili [4], Walter Aureli [3], Alfredo Miccheli [2,4*]

§ equally contributing to the article

[1] Department of Chemistry, Sapienza University of Rome, P.le Aldo Moro 5, 00185 Rome, Italy; fabio.sciubba@uniroma1.it (FS), ottavia.gianpaoili@uniroma1.it (OG), giorgia.conta@uniroma1.it (GC), giorgio.capuani@uniroma1.it (GGC), alberta.tomassini@uniroma1.it (AT);

[2] NMR-based metabolomics laboratory (NMLab), Sapienza University of Rome, P.le Aldo Moro 5, 00185 Rome, Italy

[3] R&D, Aureli Mario S.S. Agricola, Via Mario Aureli 7, 67050 Ortucchio (Aq), Italy; r.d@aurelimario.com (GG), produzione@aurelimario.com (WA)

[4] Department of Environmental Biology, Sapienza University of Rome, P.le Aldo Moro 5, 00185 Rome, Italy; elisa.brasili@uniroma1.it (EB), alfredo.miccheli@uniroma1.it (AM)

* Corresponding Author: Prof. Alfredo Miccheli, Department Of Environmental Biology, Sapienza University of Rome, P.le Aldo Moro 5, 00185 Rome, Italy; e-mail: alfredo.miccheli@uniroma1.it

Received: date; Accepted: date; Published: date

Extended Material and Methods

1) Sample Preparation

A total of 1,5 g of grated RBs for each harvest time and 1,5 ml of juice were extracted following a modified Bligh-Dyer protocol [18]. Each aliquot of grated root was placed in a mortar, grounded in liquid nitrogen, and a cold mixture composed of chloroform, methanol, and water in a 2:2:1 proportion was added. After an overnight incubation at 4°C, the samples were centrifuged for 25 min at 4°C with a rotation speed of 10,000 rpm on an Itettich Zentrifugen centrifuge (Germany). The upper hydrosoluble phase and the lower lipophilic phase were carefully separated and dried under a gentle flow of nitrogen. The hydrophilic phase was resuspended in a mixture of D_2O/MeOD in a ratio of 2:1 containing 3-(trimethylsilyl)-propionic-2,2,3,3-d_4 acid sodium salt (TSP)) 2 mM as an internal chemical shift and concentration standard. All solvents and standards were purchased from Sigma Aldrich (St. Louis, MO, USA).

2) NMR Experiments

All spectra were recorded at 298 K on a Bruker AVANCE III spectrometer (Bruker BioSpin, Karlsruhe, Germany), equipped with a Bruker multinuclear z-gradient inverse probe-head operating at the proton frequency of 400.13 MHz. ^{1}H NMR spectra were acquired employing the *presat* pulse sequence for solvent suppression with 64 transients, a spectral width of 6009.13 Hz and 64K data points for an acquisition time of 5.45 s. The employed pulse length was 14.5 μs corresponding to a flip angle of 90° while the *presat* time length was of 2 s. The recycle delay was set to 6.55 s to achieve complete resonance relaxation between successive scans.

Bidimensional NMR experiments, ^{1}H–^{1}H TOCSY, ^{1}H–^{13}C HSQC, and ^{1}H–^{13}C HMBC, were performed on a few samples in order to allow univocal signal assignment and to identify the less superimposed resonances for each molecule to achieve the best selectivity and accuracy of quantification.

TOCSY experiments were acquired with spectral width of 6009.15 Hz in both dimensions, a data matrix of 8 K × 256 points, mixing time of 110 ms and relaxation delay of 2 s.
HSQC experiments were performed with a spectral width of 6009.15 and 20000 Hz for the proton and carbon respectively, a data matrix of 8 K × 256 points, an average $^{1}J_{C-H}$ of 145 Hz and recycle delay of 2 s.
Several HMBC spectra were acquired with a spectral width of 6009.15 and 25000 Hz for the proton and carbon respectively, a data matrix of 8 K × 256 points, long-range constants $^{n}J_{C-H}$ of 4, 8 and 12 Hz and recycle delay of 2 s.
Monodimensional ^{1}H spectra were analyzed with 1D NMR Manager software ver. 12 (ACD/Labs, Toronto, Canada): FIDs underwent exponential multiplication (LB = 0.09 Hz), Fourier transformed, phase and baseline corrected. Baseline correction was automatically performed through the software built-in "FID Reconstruction" function that eliminates the broad baseline components in the time domain before Fourier Transform. Bidimensional spectra were processed with Bruker Topspin ver. 2.1. The assignment of the resonances was performed by the analysis of ^{1}H and ^{13}C NMR characteristics and cross-correlated signals in 2D spectra and by comparison with the literature compilations.
Only the molecules univocally identified were considered for the study, and their quantification was performed by integration of their NMR signals.
The selected resonances were manually integrated and then normalized for the number of protons generating the signal. These values were compared with the normalized integral of TSP (internal concentration standard) and the obtained concentrations were further normalized for the fresh weight of beets or to the juice volume. Quantities were finally expressed as mg/100 g for raw beets and as mg/100 ml for juices.

3) Statistical Analysis
Multivariate PCA was performed on the data matrix with the Unscrambler ver. 10.5 software (Camo Software AS, Oslo, Norway). Data were mean centered since the variables with the largest response could dominate the PCA, and then autoscaled to equalize the importance of the variation of each variable. The model was validated through Full Cross Validation employing the software routine.
Univariate one-way ANOVA was performed with SigmaPlot 14.0 software (Systat Software Inc., San Jose, California). The Shapiro-Wilk's test was performed on each variable to assess data normality prior to one-way ANOVA. For the ANOVA-positive variables, a Holm-Sidak all pairwise multiple comparison test was applied to determine which categories were discriminated by these metabolites ($p<0.05$).

4) Soil parameters

Table S1. Soil chemical physical parameters.

Parameter	Mean ± SD
pH	7.9 ± 0.1
Total nitrogen (g/Kg)	1.8 ± 0.3
Associated phosphorus (ppm)	255.7 ± 51.0
Exchangeable K	232.1 ± 60.1
Exchangeable Ca	3530.4 ± 601.2
Exchangeable Mg	254.7 ± 31.2
C/N	8.5 ± 0.5

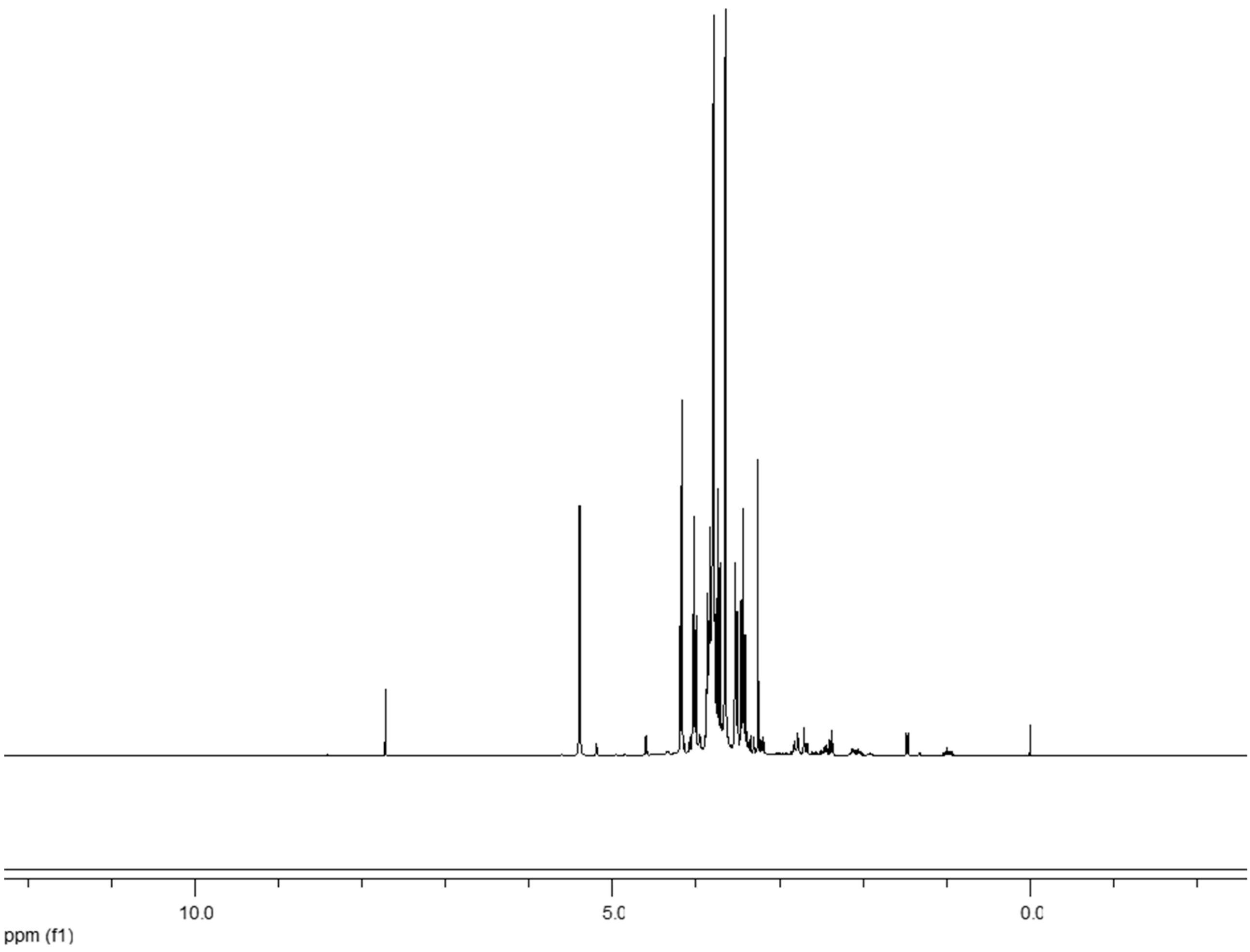

Figure S1. ^{1}H Spectrum of red beetroot hydroalcoholic extract.

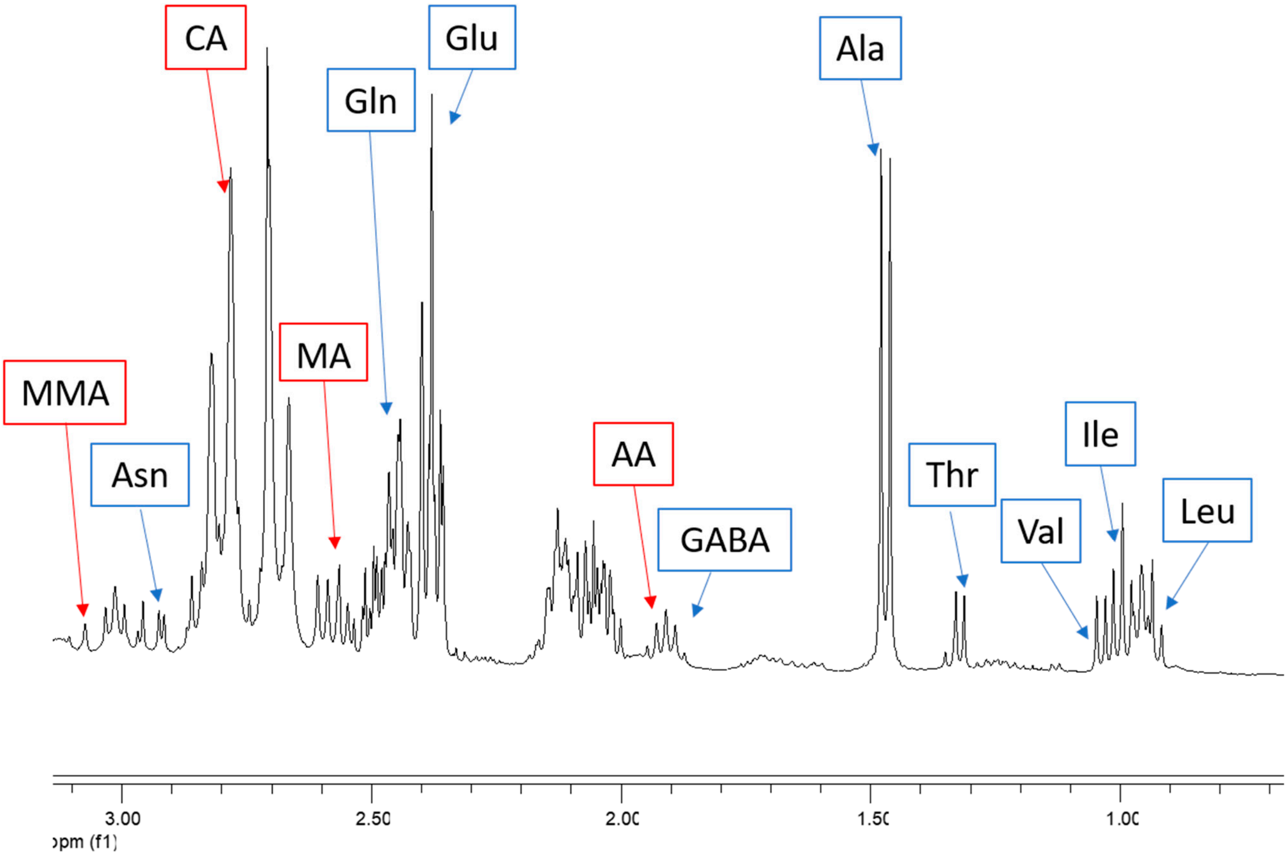

Figure S2. ^{1}H Spectrum of red beetroot hydroalcoholic extract, 0.7-3.1 ppm. Molecule abbreviations are reported in Table S1.

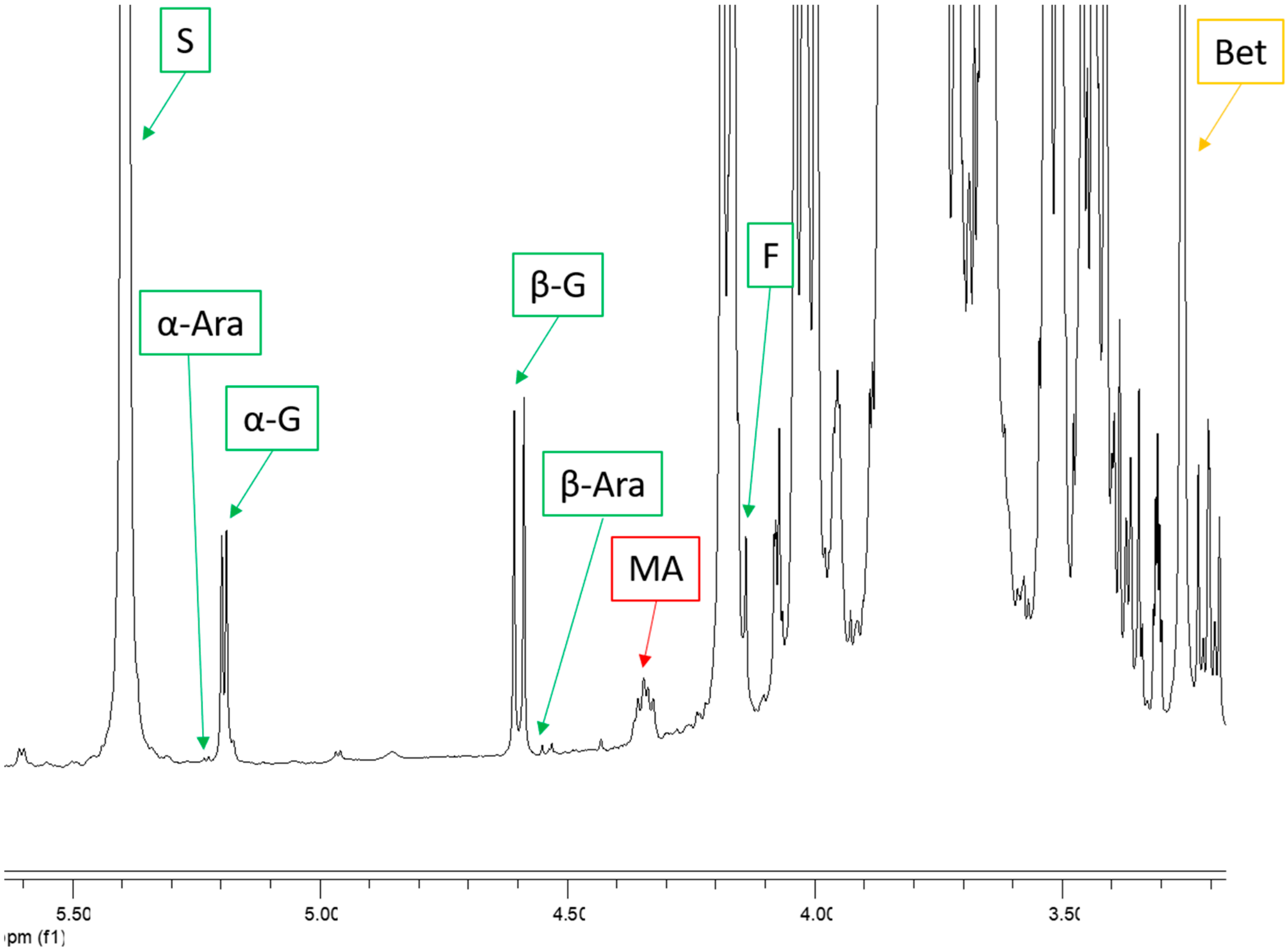

Figure S3. ^{1}H Spectrum of red beetroot hydroalcoholic extract, 3.1-5.5 ppm. Molecule abbreviations are reported in Table S1.

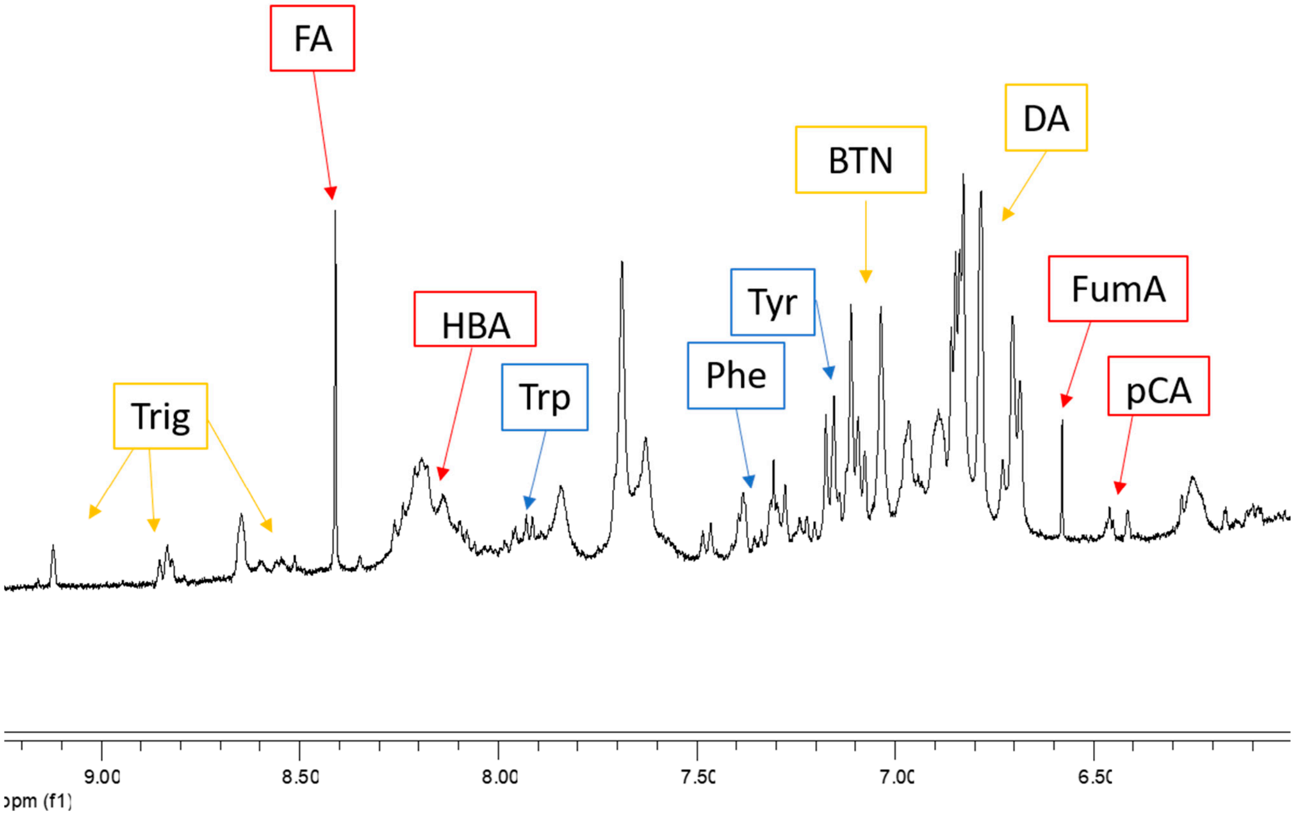

Figure S4. ^{1}H Spectrum of red beetroot hydroalcoholic extract, 6.0-9.2 ppm. Molecule abbreviations are reported in Table S1.

Table S2: Resonance assignment of red beetroot hydroalcoholic extract; s: singlet, d: doublet, t: triplet, q: quartet, dd: doublet of doublets, m: multiplet, bs: broad singlet

Compound	Assignment	^{1}H δ (ppm)	Multiplicity	^{13}C δ (ppm)
**Organic Acid**				
**Acetate (AA)**	**CH_3**	**1.92**	**s**	25.98
**Citrate (CA)**	**α,γ-CH**<br>α′,γ′-CH	**2.67**<br>2.71	**d**<br>d	44.77<br>44.77
**Malonate (MMA)**	**CH_2**	**3.08**	**s**	50.53
**Formate (FA)**	**CH**	**8.46**	**s**	171.90
**Fumarate (FumA)**	**CH=CH**	**6.51**	**s**	137.94
Lactate	CH_3<br>CH	1.32<br>4.11	d<br>q	22.95<br>77.04
**Malate (MA)**	**α-CH**<br>β-CH<br>β′-CH	**4.31**<br>2.69<br>2.38	**dd**<br>dd<br>dd	69.33<br>40.86<br>40.86
**p-Hydroxybenzoate (HBA)**	**CH-2,6**<br>CH-3,5	**7.80**<br>6.84	**d**<br>d	132.96<br>116.21
Gallic acid	CH-2,6	7.05	s	112.2
p-Hydroxycoumarate	α-CH<br>β-CH<br>CH-2,6<br>CH-3,5	6,36<br>7,49<br>6,80<br>7,51	d<br>d<br>m<br>m	124.2<br>143.5<br>118.6<br>132.3
**Succinate (SA)**	**α,β-CH_2**	**2.42**	**s**	36.31
**Amino acids**				
**Alanine (Ala)**	**β-CH_3**<br>α-CH	**1.49**<br>3.80	**d**<br>q	19.05<br>53.56
**Asparagine (Asn)**	β′-CH<br>**β-CH**<br>α-CH	2.86<br>**2.89**<br>4.01	dd<br>**dd**<br>m	37.44<br>37.44<br>54.09
**γ-aminobutyrate (GABA)**	β-CH_2<br>γ-CH_2<br>**α-CH_2**	1.95<br>2.30<br>**3.01**	t<br>m<br>**t**	26.38<br>37.06<br>42.21
**Glutamine (Gln)**	γ-CH_2<br>**β-CH_2**<br>α-CH	2.11<br>**2.45**<br>3.81	m<br>**m**<br>m	29.31<br>34.02<br>57.19
**Glutamate (Glu)**	γ-CH_2<br>**β-CH_2**<br>α-CH	2.09<br>**2.34**<br>3.74	m<br>**m**<br>m	28.9<br>36.32<br>56.79
**Isoleucine (Ile)**	δ-CH_3<br>**γ-CH_3**	0.95<br>**1.02**	t<br>**d**	13.85<br>17.38

	γ′-CH	1.25	m	27.01
	γ′′-CH	1.49	m	27.01
	β-CH	1.99	m	38.71
	α-CH	3.69	m	63.04
**Leucine (Leu)**	**δ,δ′-CH$_3$**	**0.97**	**m**	23.85, 24.59
	γ-CH	1.72	m	26.81
	β-CH$_2$	1.73	m	42.60
	α-CH	3.74	m	56.21
**Phenylalanine (Phe)**	CH-2,6	**7.32**	**d**	130.3
	CH-4	**7.38**	**d**	128.6
	**CH-3,5**	**7.42**	**d**	130.3
	β-CH$_2$	3.27	m	37.1
	α-CH	3.98	dd	56.8
**Threonine (Thr)**	**γ-CH$_3$**	**1.32**	**d**	22.15
	α-CH	3.60	m	63.46
	β-CH	4.27	m	68.94
**Tryptophan (Trp)**	CH-5	7.20	t	124.9
	CH-6	7.27	t	127.9
	**CH-7**	**7.53**	**d**	114.7
	CH-4	7.73	d	121.2
**Tyrosine (Tyr)**	CH-2,6	7.17	d	130.0
	**CH-3,5**	**6.89**	**d**	117.0
	β-CH$_2$	3.15	dd	37.1
	α-CH	3.93	dd	56.8
**Valine (Val)**	γ-CH$_3$	0.99	d	19.41
	**γ′-CH$_3$**	**1.05**	**d**	20.75
	β-CH	2.29	m	31.89
	α-CH	3.62	m	63.36
**Carbohydrates**				
α-Arabinose	CH-1	5.23	d	95.3
	CH-3	3.98	m	71.4
	CH-4	3.86	m	71.2
	CH-2	3.81	m	71.5
	CH$_2$-5	3.63-4.09	m	65.2
β-Arabinose	CH-1	4.52	d	99.6
	CH-4	3.92	m	71.1
	CH-3	3.65	m	75.2
	CH-2	3.50	m	74.8
	CH$_2$-6	3.67-3.88	m	69.2
**Fructose (F)**	CH-1	3.69	m	62.94
	C-2	\	\	104.23
	**CH-3**	**4.22**	**d**	77.24
	CH-4	4.06	m	75.04
	CH-5	3.90	m	83.37
	CH-6	3.82	m	63.88
**α-Glucose (α-G)**	**CH-1**	**5.25**	**d**	93.10

	CH-2	3.55	m	72.49
	CH-3	3.72	m	73.84
	CH-4	3.42	m	70.67
	CH-5	3.84	m	72.52
	CH_2-6	3.73, 3.90	m	96.97
**β-Glucose (β-G)**	**CH-1**	**4.69**	**d**	96.97
	CH-2	3.26	m	75.17
	CH-3	3.50	m	76.84
	CH-4	3.42	m	70.70
	CH-5	3.48	m	74.57
	CH_2-6	3.74, 3.91	m	61.80
**Myo Inositol (Myo)**	**CH-1**	**4.05**	**dd**	74.93
	CH-2,2′	3.61	m	75.13
	CH-3,3′	3.53	m	73.97
	CH-4	3.27	dd	76.98
**Sucrose (S)**	**G CH-1**	**5.42**	**d**	93.22
	CH-2	3.59	m	72.11
	CH-3	3.79	m	73.54
	CH-4	3.48	m	70.26
	CH-5	3.85	m	73.38
	CH_2-6	3.82	m	61.18
	F CH_2-1′	3.69	m	62.44
	C-2	\	\	104.85
	CH-3′	4.22	m	77.45
	CH-4′	4.06	m	75.04
	CH-5′	3.90	m	82.44
	CH_2-6	3.82	m	63.38
**Miscellaneous Metabolites**				
**Betaine (Bet)**	**N-$(CH_3)_3$**	**3.26**	**s**	55.95
	CH_2	3.84	s	68.58
**Betanin (BTN)**	**CH-4**	**7.04**	**bs**	102.54
	CH-7	7.11	bs	116.91
**Choline (Chn)**	**N-$(CH_3)_3$**	**3.21**	**s**	56.74
	CH_2	3.51	t	70.20
	CH_2	4.07	t	57.78
**Dihydroxyacetone (DHA)**	**CH_2**	**4.43**	**s**	66.2
**(Dimethylamine) DMA**	**CH_3, CH_3′**	**2.50**	**s**	37.40
**Dopamine (DA)**	CH-2	6.87	m	119.62
	CH-4	6.85	m	119.58
	**CH-5**	**6.73**	**pd**	123.83
	α-CH_2	3.22	t	43.68
	β-CH_2	2.88	t	36.34
**Trigonelline (Trg)**	**CH-1**	**9.11**	**s**	148.4
	CH-3	8.83	m	148.71
	CH-4	8.11	m	130.58

	CH-5<br>CH_3	8.83<br>4.43	m<br>s	148.71<br>51.07
